# Automatic Multi-functional Integration Program (AMFIP) towards all-optical mechano-electrophysiology interrogation

**DOI:** 10.1371/journal.pone.0266098

**Published:** 2022-07-28

**Authors:** Qin Luo, Justin Zhang, Miao Huang, Gaoming Lin, Mai Tanaka, Sharon Lepler, Juan Guan, Dietmar Siemann, Xin Tang

**Affiliations:** 1 Department of Electrical and Computer Engineering, Herbert Wertheim College of Engineering, University of Florida, Gainesville, Florida, United States of America; 2 Department of Electrical and Computer Engineering, University of California, Santa Barbara, California, United States of America; 3 Department of Mechanical and Aerospace Engineering, Herbert Wertheim College of Engineering, UF, Gainesville, Florida, United States of America; 4 Department of Radiation Oncology, College of Medicine, University of Florida, Gainesville, Florida, United States of America; 5 UF Health Cancer Center, University of Florida, Gainesville, Florida, United States of America; 6 Department of Physics, College of Liberal Arts and Sciences, University of Florida, Gainesville, Florida, United States of America; 7 Department of Anatomy and Cell Biology, College of Medicine, University of Florida, Gainesville, Florida, United States of America; Mohanlal Sukhadia University, INDIA

## Abstract

Automatic operations of multi-functional and time-lapse live-cell imaging are necessary for the biomedical science community to study active, multi-faceted, and long-term biological phenomena. To achieve automatic control, most existing solutions often require the purchase of extra software programs and hardware that rely on the manufacturers’ own specifications. However, these software programs are usually non-user-programmable and unaffordable for many laboratories. To address this unmet need, we have developed a novel open-source software program, titled Automatic Multi-functional Integration Program (AMFIP), as a new Java-based and hardware-independent system that provides proven advantages over existing alternatives to the scientific community. Without extra hardware, AMFIP enables the functional synchronization of the μManager software platform, the Nikon NIS-Elements platform, and other 3rd party software to achieve automatic operations of most commercially available microscopy systems, including but not limited to those from Nikon. AMFIP provides a user-friendly and programmable graphical user interface (GUI), opening the door to expanding the customizability for myriad hardware and software systems according to user-specific experimental requirements and environments. To validate the intended purposes of developing AMFIP, we applied it to elucidate the question whether single cells, prior to their full spreading, can sense and respond to a soft solid substrate, and if so, how does the interaction depend on the cell spreading time and the stiffness of the substrate. Using a CRISPR/Cas9-engineered human epithelial Beas2B (B2B) cell line that expresses mNeonGreen2-tagged mechanosensitive Yes-associated protein (YAP), we show that single B2B cells develop distinct substrate-stiffness-dependent YAP expressions within 10 hours at most on the substrate, suggesting that cells are able to sense, distinguish, and respond to mechanical cues prior to the establishment of full cell spreading. In summary, AMFIP provides a reliable, open-source, and cost-free solution that has the validated long-term utility to satisfy the need of automatic imaging operations in the scientific community.

## Introduction

**Fig 1:**
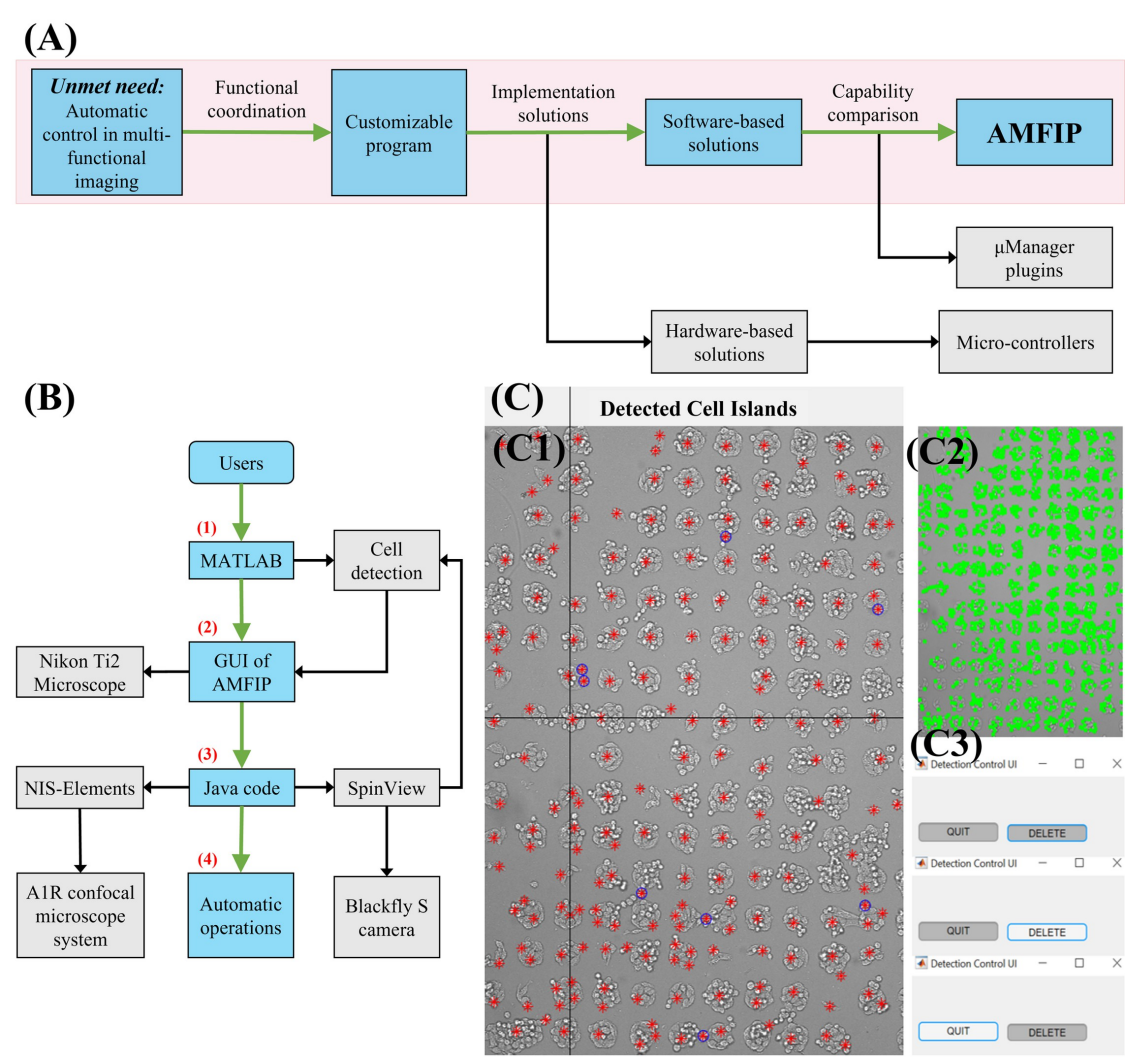
The design of automatic multi-functional integration program (AMFIP). (**A**) Software-based AMFIP is designed and developed to achieve customizable and automatic control of generic microscopy systems. This capability is needed in many research laboratories. AMFIP enables automatic, customizable, and hardware-independent control (in blue) of multi-functional imaging conditions to acquire spatial-temporal biological information. In contrast, other software-based solutions (in grey), such as μManager plugins, and hardware-based solutions, such as micro-controllers, only control limited hardware and thus provide restricted functions. (**B**) The Workflow of implementing AMFIP in a typical experiment. (1) A custom MATLAB program assists in detecting the cells of interest and generates a coordinate list of selected FOVs. (2) The GUI of AMFIP reads the coordinates to guide the movement of the XY motor-stage. Users input pre-defined experimental parameters and initiate μManager to modulate the Nikon Ti2-E microscope. (3) Java code in AMFIP activates Elements and SpinView to manipulate the A1R confocal microscope system and Blackfly S camera. The MATLAB program reads the latest bright-field image to update the coordinates of current FOVs. (4) AMFIP automatically conducts these operations in sequence for multi-functional imaging. (**C**) A representative view of MATLAB operation interface for automatic cell detection and stage-drift prevention. (**C1**) The Interface of MATLAB to edit marks of detected cell islands. Red marks indicate the centroids of detected cell islands while blue circles denote unwanted marks. The mis-match rate (number of marks that are not cell divided by number of overall marks) is 4.66% ± 0.82%. (**C2**) Patterns of detected cells. The detected cells are marked with green rectangles. (**C3**) Detection control with “DELETE” and “QUIT” buttons. The button in grey indicates that its statement is inactivated. By clicking the button, its function is activated.

Automatic operations of multi-functional and time-lapse live-cell imaging are essential for the biomedical science community that explore dynamic, multi-faceted, and long-term biological questions [1–6]. Successful automatic operations of myriad custom experiments require streamlined functional coordinations of multiple microscope hardware and imaging software systems that are produced by different manufacturers of optoelectronic systems. However, most manufacturers usually provide their own hardware-specific drivers and software. Despite their high price, these expensive drivers and software are often non-user-programmable and incompatible with additional 3^rd^ party hardware, limiting the full utilization of hardware functionalities and the necessary coordination between different devices [7–9]. To address this unmet need in the scientific community, we have developed a novel open-source software-based automation program, titled Automatic Multi-functional Integration Program (AMFIP), using Java programming language (**Fig 1A**). Compared with existing alternatives available to researchers, AMFIP applies a common programming language Java to coordinate multiple software and overcomes the challenges of developing an individual driver for every new hardware. Most newly developed optoelectronic devices that come with official controlling software can be directly controlled by our program through the coordination of AMFIP with the related official controlling software. Therefore, our program provides advantages to the scientific community by functioning as a hardware-independent controlling hub with the validated long-term utility, enabling the functional coordination of multiple hardware and software systems to achieve automatic multi-functional and time-lapse data acquisition on most commercially available imaging systems.

AMFIP is developed based on the μManager software platform and entails multiple advantages (**Table 1**). First, AMFIP achieves software-based automation without adding any extra hardware. To achieve automatic operations, some existing solutions require extra single-board microcontrollers to coordinate multiple hardware and software systems. These microcontrollers generate analog and digital signals to modulate optoelectronic devices. For example, the NI data acquisition card (DAQ; National Instrument Corp.) is utilized to control the frame acquisition of the camera and switch of the laser channels [10]. An Arduino-based system is applied to modulate selective-plane illumination microscopy (SPIM) [11]. However, these hardware-based solutions need additional purchases of expensive adaptive drivers at prices around the $1000s for some optoelectronic devices, such as the Nikon A1R controller. Our hardware-independent AMFIP program avoids such expenses by implementing a home-built Java-based script coordinating all devices through software communications alone. Traditionally, to transmit modulatory signals from the NI digital-analog converters (DAC) card into some optoelectronics hardware, particular physical ports are needed, such as a Communication (COM) port or Peripheral Component Interconnect (PCI) port. However, these ports are not always present in many commercial devices, such as the Nikon LU-N4 laser units used by many research laboratories. Thus, additional purchases are needed for researchers to control the new hardware components that contain these ports [12]. AMFIP bypasses these hardware constraints and accomplishes automatic modulations by leveraging the software communications between the Nikon NIS-Elements software platform (Elements) that exclusively controls Nikon’s hardware and the hardware equipment from 3^rd^ party manufacturers.

**Table 1:** Comparison between AMFIP and other existing software- and hardware-based solutions.

	Software-based solutions			Hardware-based solutions	
	AMFIP (Developed in this work)	MultiFRET [13]	EMU [14]	μSPIM [12]	mmSIM [10]
**Key hardware used for automatic operations**	Nikon Ti2-E inverted microscope (μManager-supported hardware) Blackfly camera (μManager-supported hardware) Nikon A1R confocal microscope system (non-μManager-supported hardware)	Nikon TE2000 microscope (μManager-supported hardware)	Only μManager-supported hardware	Laser from Omicron and Cobolt (μManager-supported hardware) E-665 Piezo Amplifier (μManager-supported hardware) ORCA-flash Camera (non-μManager-supported hardware)	Olympus IX71 microscope (μManager-supported hardware)
	Purchase of additional hardware is not needed			Purchase of NI DAQ card (PCI/COM ports required) is needed	
**Software available for coordination**	μManager, Nikon NIS-Elements, SpinView	Only μManager			
**Achieved automatic functionalities**	Modulation of motor-stage, objectives, filter wheel, light path, and laser Bright-field imaging and confocal microscopy imaging (time-lapse or z-stack) Fluorophore bleaching	High-throughput Förster Resonance Energy Transfer (FRET) image acquisition	Modulation of filter wheel and laser. Localization microscopy imaging (time series or z-stack)	Modulation of motor-stage. Selective Plane Illumination Microscopy Imaging	Modulation of motor-stage, laser, and filter wheel. Structured illumination microscopy (SIM) imaging

AMFIP coordinates μManager with 3rd party software, such as Elements and SpinView, to manipulate both μManager-supported and non-μManager-supported hardware, such as Nikon A1R confocal microscope system. In contrast, other software-based solutions, such as MultiFRET and EMU, modulate limited μManager-supported hardware, e.g., Nikon TE2000 microscope. AMFIP does not require the purchase of additional hardware. Most hardware-based solutions, such as μSPIM and mmSIM, require an NI DAQ card to control.

Second, compared to other existing solutions, AMFIP supports a broader range of hardware, including but not limited to μManager-supported hardware. As an open-source software package, μManager has been applied to manipulate optoelectronic devices [15–19]. Researchers have developed myriad user-defined μManager plugins to achieve specific tasks. However, due to the rapid upgrades of microscopy equipment in the market, most existing μManager plugins lack development speedy enough to provide sufficient compatibility with the latest equipment. For example, a recently developed μManager plugin, MultiFRET, can achieve automatic acquisition and analysis of fluorescent images but relies on μManager-supported instruments [13]. Due to the same restriction, another newly developed plugin, Easier Micro-Manager User (EMU), can offer only limited functions to control μManager-supported optical hardware, e.g., modulation of laser and filter wheels, and acquisition processes, e.g., 2D time series or 3D z-stack imaging [14]. AMFIP is compatible with non-μManager-supported instruments, providing more choices on new hardware to be included in microscope systems for a more complex automatic process. We have demonstrated that AMFIP enables automatic operations such as modulations of the Nikon laser channels and multi-functional imaging, which have been previously unattainable by μManager alone, through the coordination with Nikon’s official controlling software Elements (Results) [15].

**Fig 2:**
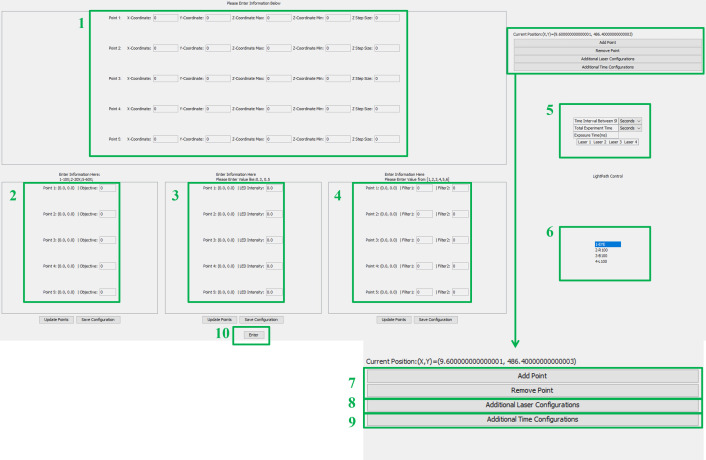
The graphical user interface (GUI) of AMFIP. The all-in-one GUI of AMFIP enables users to program all experimental parameters and essential functions according to users’ specific experimental requirements and environment. Users are allowed to (1) Input X, Y, Z coordinates for each FOV; (2) Specify the types of microscope objective for each FOV; (3) Specify the intensity of DiaLamp for each FOV; (4) Specify desired filter cubes for each FOV; (5) Define the universal time and laser configurations for each FOV in the experiment; (6) Switch to different light path; (7) Add or remove points; (8) Open up the window for additional laser configuration; (9) Open up the window for additional time configuration; and (10) Start the specified experimental procedure.

Third, Java-based AMFIP enables user-programmable and automatic operations. Specifically, AMFIP has a user-friendly GUI and is programmable to perform operations that meet the experiment-specific requirements (**Fig 2**). Prior to experiments, researchers can flexibly specify the functions needed for their experiments and input the required parameters. Next, AMFIP coordinates all hardware and software involved and executes the specified tasks automatically in the experiments (**Fig 1B**). In case unexpected accidents occur during the course of the experiments, researchers can flexibly pause the current task without any loss of acquired data and resume the experiments any time after clearance of the accidents. For comparison, an existing MATLAB-based GUI [20] can automatically accomplish an 18-hour data acquisition, but it cannot pause the experiment in case of unexpected accidents. Instead, AMFIP achieves automation of the entire system while providing users with safe and flexible control. These features are desirable for both new and experienced users.

**Fig 3:**
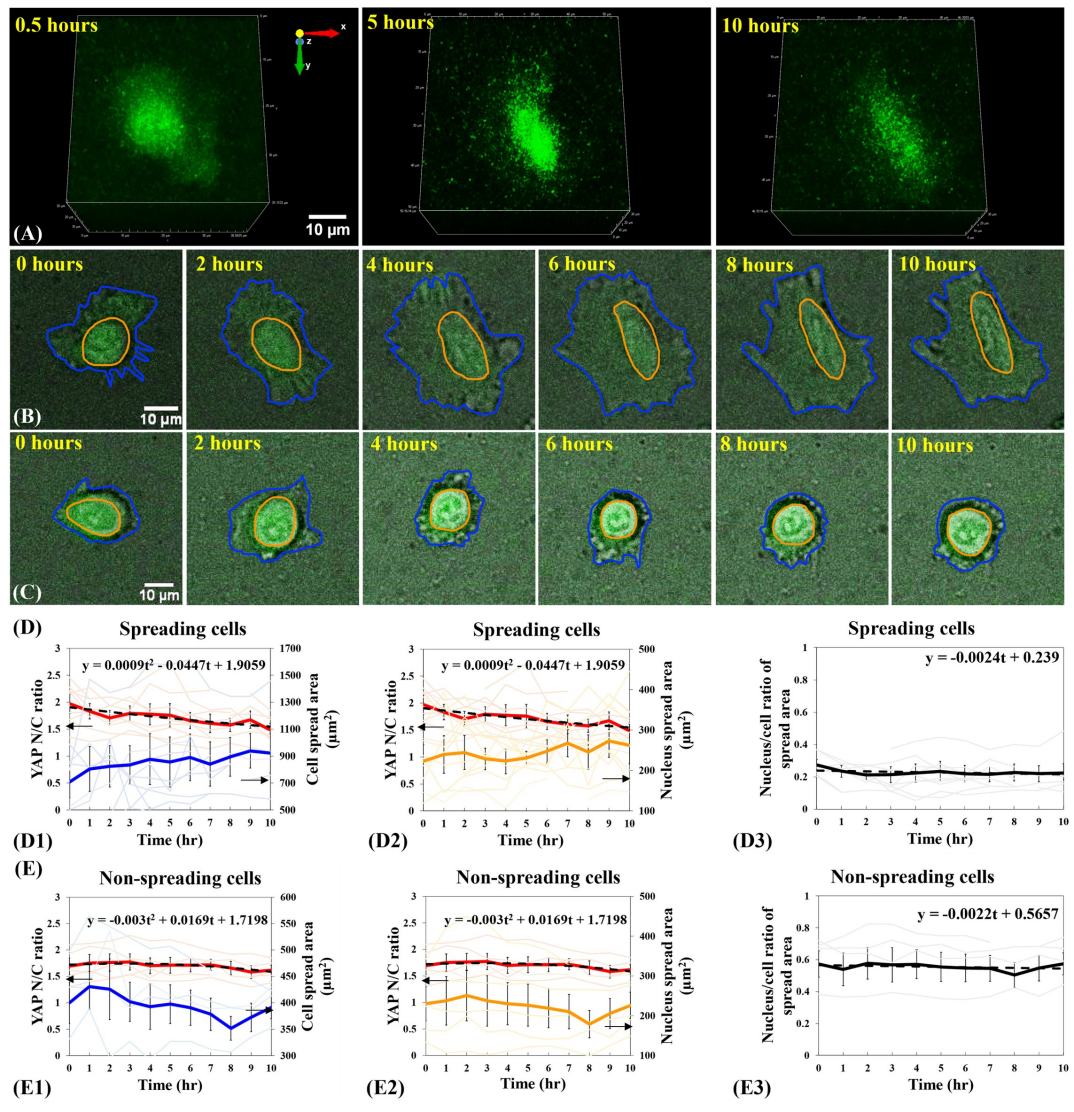
Relationship between YAP N/C ratio and cell spreading states of single B2B cells on 2-kPa hydrogels substrates. (**A**) 3D fluorescent confocal YAP image stack of a single spreading B2B cell on 2-kPa substrate at 0.5^th^, 5^th^, and 10^th^ hour. Notably, YAP intensity in nucleus decreases with respect to that in cytoplasm and the YAP distribution becomes homogenous during the process of cell spreading. (**B**) 10-hr time-lapse image stack that contains overlapped fluorescent YAP images and bright-field cell images of a single B2B cell spreading on 2-kPa hydrogels. Note: The shapes of the cell (in blue contour) and nucleus (in orange contour) transform from roundness to flatness. YAP N/C ratio decreases from 1.93 at 0th hour to 1.40 at 10th hour. (**C**) 10-hr time-lapse image stack of a non-spreading single cell. Note: The shape of the cell maintains rounded. YAP N/C ratio changed from 1.62 at 0th hour to 1.59 at 10th hour. (**D**) YAP N/C ratio versus nucleus/cell-body area of spreading cells (n = 10). The average YAP N/C ratio (red bold line; n = 10) changed from 1.97 ± 0.17 to 1.49 ± 0.12 (p-value = 0.0022**; **D1**). The average cell area (blue bold line; n = 10) increased from 708.96 ± 118.71 μm^2^ to 922.30 ± 147.73 μm^2^ (p-value = 0.0920 (ns); **D1**) and the average nucleus area (orange bold line; n = 10) changed from 222.70 ± 41.35 μm^2^ to 262.37 ± 45.64 μm^2^ (p-value = 0.3220 (ns); **D2**). The average nucleus-spread-area/cell-spread-area ratio changed from 0.27 ± 0.03 to 0.22 ± 0.06 over time (p-value = 0.2422 (ns))with a trend-line slope of -0.0024 (**D3**). (E) YAP N/C ratio versus nucleus/cell-body area of non-spreading cells (n = 5). The average YAP N/C ratio (red bold line) changed from 1.69 ± 0.16 to 1.62 ± 0.09 (p-value = 0.6741 (ns); **E1**), and the average cell area (blue bold line) changed from 399.49 ± 38.74 μm^2^ to 391.24 ± 21.30 μm^2^ (p-value = 0.8400 (ns); **E1**). The average nucleus area (orange bold line) changed from 230.02 ± 43.03 μm^2^ to 225.83 ± 33.60 μm^2^ (p-value = 0.9377 (ns); **E2**) The average nucleus-spread-area/cell-spread-area ratio maintained at 0.57 ± 0.07 (p-value = 0.9908 (ns)), with a trend-line slope of -0.0022 (**E3**). All p-values are indicated according to the Michelin guide scale (p ≤ 0.001: [***]; 0.001 < p ≤ 0.01: [**]; 0.01 < p ≤ 0.05: [*]; 0.05 < p: ns).

**Fig 4:**
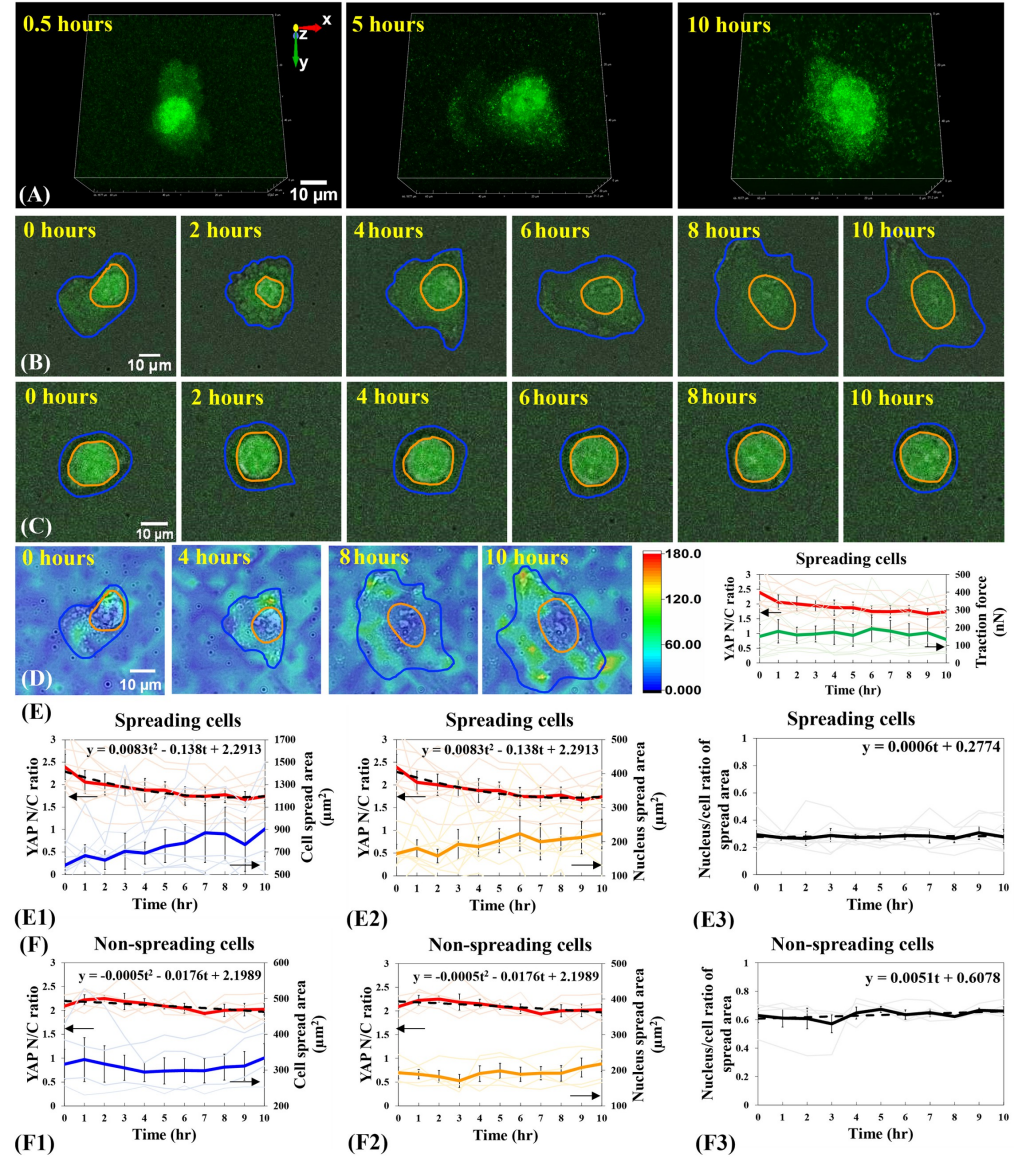
YAP N/C ratio and cell spreading states of single B2B cells on 5-kPa substrates. (A) 3D fluorescent YAP image stack of the single spreading B2B cell at 0.5^th^, 5^th^ and 10^th^ hour. Note: YAP intensity in nucleus remains higher than the intensity in cytoplasm through the process of cell spreading. (B) 10-hr time-lapse image stack of a single spreading B2B cell on 5-kPa substrates. Note: The cell body (in blue contour) and the nucleus (in orange contour) flatten down. YAP N/C ratio changes from 2.09 at 0th hour to 1.73 at 10th hour. (C) 10-hr time-lapse image stack of a non-spreading single cell. The YAP N/C ratio changed from 2.00 at 0^th^ hour to 2.09 at 10^th^ hour. (D) A filmstrip of the traction field under the single spreading cell (A) and YAP N/C ratio vs. traction as a function of time (n = 10). The magnitude of traction remains constant as the cell spreads over time. (E) YAP N/C ratio versus nucleus/cell-body area of single spreading cells (n = 10). The average YAP N/C ratio (red bold line; n = 10) changed from 2.39 ± 0.28 to 1.74 ± 0.17 (p-value = 0.0057**; E1). The average normalized cell area (blue bold line; n = 10) increased from 580.96 ± 77.79 μm^2^ to 906.39 ± 245.43 μm^2^ (p-value = 0.0610 (ns); E1) and the average normalized nucleus area (orange bold line; n = 10) augmented from 164.85 ± 27.21 μm^2^ to 223.50 ± 43.19 μm^2^ (p-value = 0.0860 (ns); E2). The average nucleus-spread-area/cell-spread-area ratio changed from 0.29 ± 0.05 to 0.28 ± 0.05 (p-value = 0.7461 (ns)), with a trend-line slope of 0.0006 (E3). (F) YAP N/C ratio versus nucleus/cell-body area of non-spreading cells (n = 5). The average YAP N/C ratio (red bold line; n = 5) changed from 2.09 ± 0.09 to 2.03 ± 0.12 (p-value = 0.6335 (ns); F1), and the average cell area (blue bold line; n = 5) increased from 316.12 ± 42.58 μm^2^ to 333.75 ± 40.37 μm^2^ (p-value = 0.7455 (ns); F1). The average normalized nucleus area (orange bold line; n = 5) rose from 192.85 ± 15.71 μm^2^ to 218.17 ± 22.10 μm^2^ (p-value = 0.3271 (ns); F2) The average nucleus-spread-area/cell-spread-area ratio changed from 0.63 ± 0.05 to 0.66 ± 0.03 (p-value = 0.5575 (ns)), with a trend-line slope of 0.0051 (F3).

**Fig 5:**
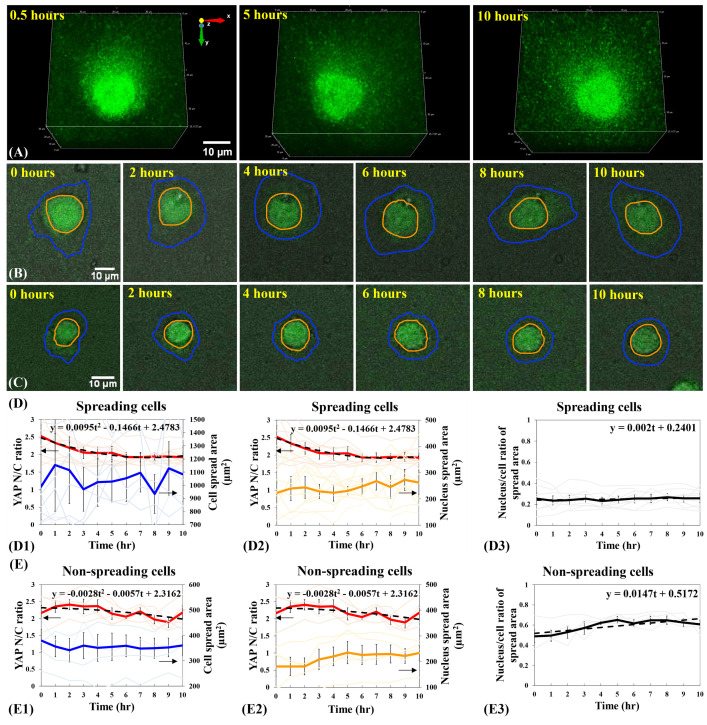
YAP N/C ratio and cell spreading states of single B2B cells on 40-kPa hydrogels substrates. (**A**) 3D fluorescent YAP image stack of the single spreading B2B cell (B) at 0.5^th^, 5^th^ and 10^th^ hour. (**B**) 10-hr time-lapse filmstrip of a single B2B cell on 40-kPa substrates during the spreading process. Note: cell flattens down and YAP N/C ratio changes from 3.14 at 0^th^ hour to 2.48 at 10^th^ hour. (**C**) 10-hr time-lapse image stack of a non-spreading single cell. The YAP N/C ratio changed from 1.95 at 0th hour to 1.89 at 10th hour. (**D**) YAP N/C ratio compared with nucleus/cell-body area of single spreading cells (n = 10). The average YAP N/C ratio (red bold line; n = 10) changed from 2.53 ± 0.32 to 1.93 ± 0.12 (p-value = 0.0118 *; **D1**). The average normalized cell area (blue bold line; n = 10) increased from 988.25 ± 247.34 μm^2^ to 1082.84 ± 207.46 μm^2^ (p-value = 0.6487 (ns); **D1**) and the average normalized nucleus area (orange bold line; n = 10) augmented from 220.70 ± 41.35 μm^2^ to 262.37 ± 45.64 μm^2^ (p-value = 0.3220 (ns); **D2**). The average nucleus-spread-area/cell-spread-area ratio changed from 0.25 ± 0.05 to 0.26 ± 0.04 (p-value = 0.9317 (ns)), with a trend-line slope of 0.002 (**D3**). (**E**) YAP N/C ratio compared with nucleus/cell-body area of non-spreading cells (n = 5). The average YAP N/C ratio (red bold line; n = 5) changed from 2.16 ± 0.23 to 2.18 ± 0.19 (p-value = 0.9440 (ns); **E1**), and the average cell area (blue bold line; n = 5) changed from 379.36 ± 43.42 μm^2^ to 360.48 ± 37.97 (ns); **E1**). The average normalized nucleus area (orange bold line; n = 5) rose from 181.47 ± 39.09 μm^2^ to 234.02 ± 28.37 μm^2^ (p-value = 0.2585 (ns); **E2**). The average nucleus-spread-area/cell-spread-area ratio changed from 0.49 ± 0.06 to 0.61 ± 0.04 (p-value = 0.1091 (ns)), with a trend-line slope of 0.0147 (**E3**).

**Fig 6:**
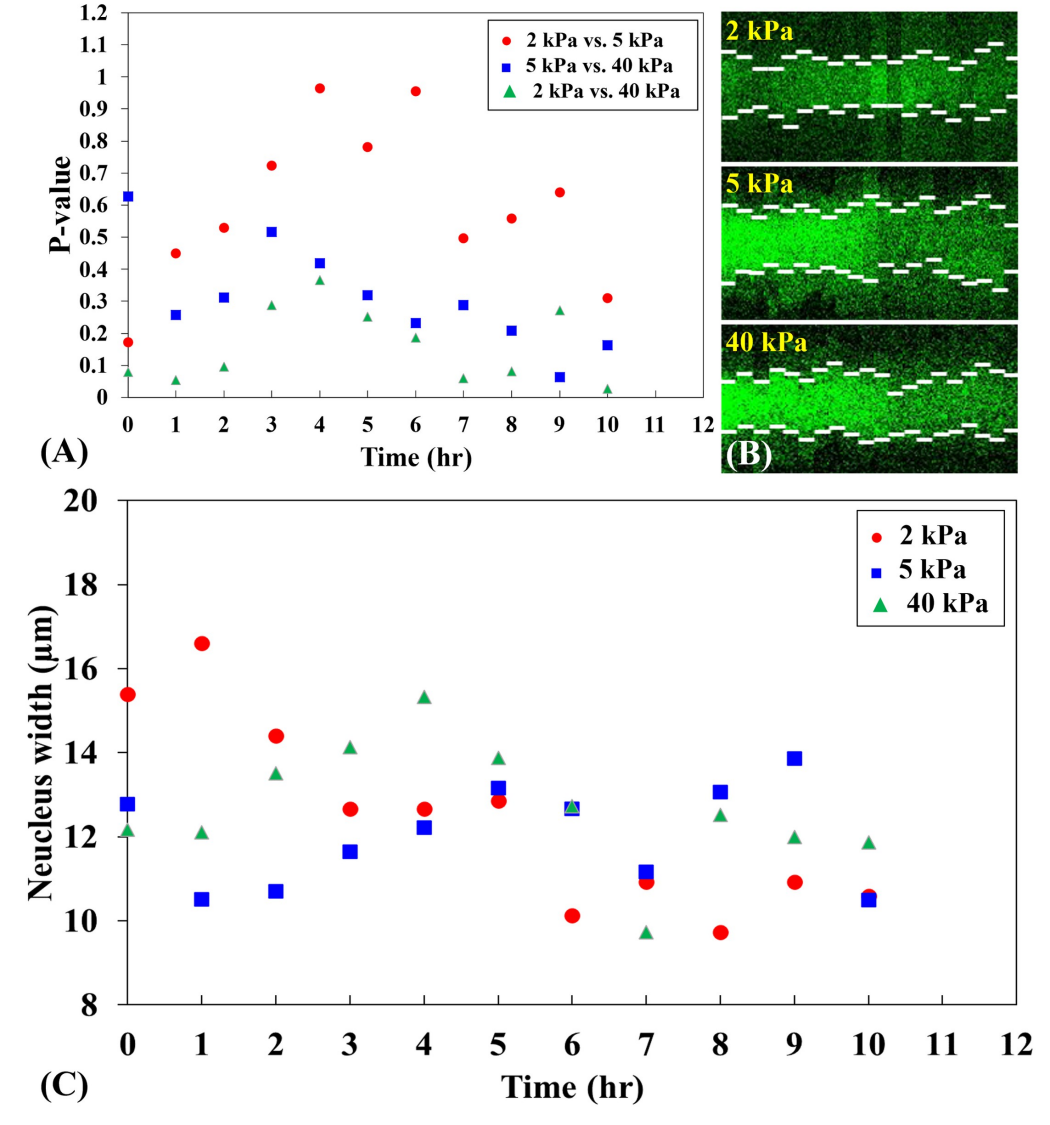
Comparison of the p-values of YAP N/C ratio on different stiffnesses of substrates during the first 10 hours of the spreading process. (**A**) The p-values of the YAP N/C ratio on 2-kPa substrates and 5-kPa substrates (red dots) increased from 0.1725 at 0^th^ hour to 0.9642 at 4^th^ hour and decreased to 0.3103 at 10^th^ hour. The p-values of the YAP N/C ratio between 5-kPa substrates and 40-kPa substrates (blue squares) showed a gradual decrease from 0.6271 at 0^th^ hour to 0.1637 at 10^th^ hour. The p-values of the YAP N/C ratio on 2-kPa substrates and 40-kPa substrates (green triangles) maintained stable at the first two hours and rose from 0.0955 at 2^nd^ hour to 0.3668 at 4^th^ hour. Next, the p-value declined to 0.0595 at 7^th^ hour and increased to 0.2727 at 9^th^ hour. At 10^th^ hour, the p-value stayed at 0.0262 (*). Generally, when the difference of the two stiffnesses being compared is small (i.e., 2 kPa vs. 5 kPa), the p-value of the YAP N/C ratio is relatively larger over time. As the difference of the two stiffnesses increases (i.e., 2 kPa vs. 40 kPa), the p-value of the YAP N/C ratio declines. (**B**) Kymographs of single spreading B2B cells on 2-kPa, 5-kPa and 40-kPa substrates during the first 10 hours of the spreading process. The change of the position of the short white lines (time interval: 0.5 hrs.) on the graphs shows fluctuations of the nucleus area and fluorescence as cells are spreading. (**C**) The nucleus width of the single spreading B2B cells from (**B**) fluctuates over time. The fluctuation of the nucleus area changes the YAP density in nucleus, which may result in a fluctuation of YAP N/C ratio. This fluctuation of the YAP N/C ratio is reflected in the three groups of p-values in (**A**), which show fluctuating trends over time.

**Fig 7:**
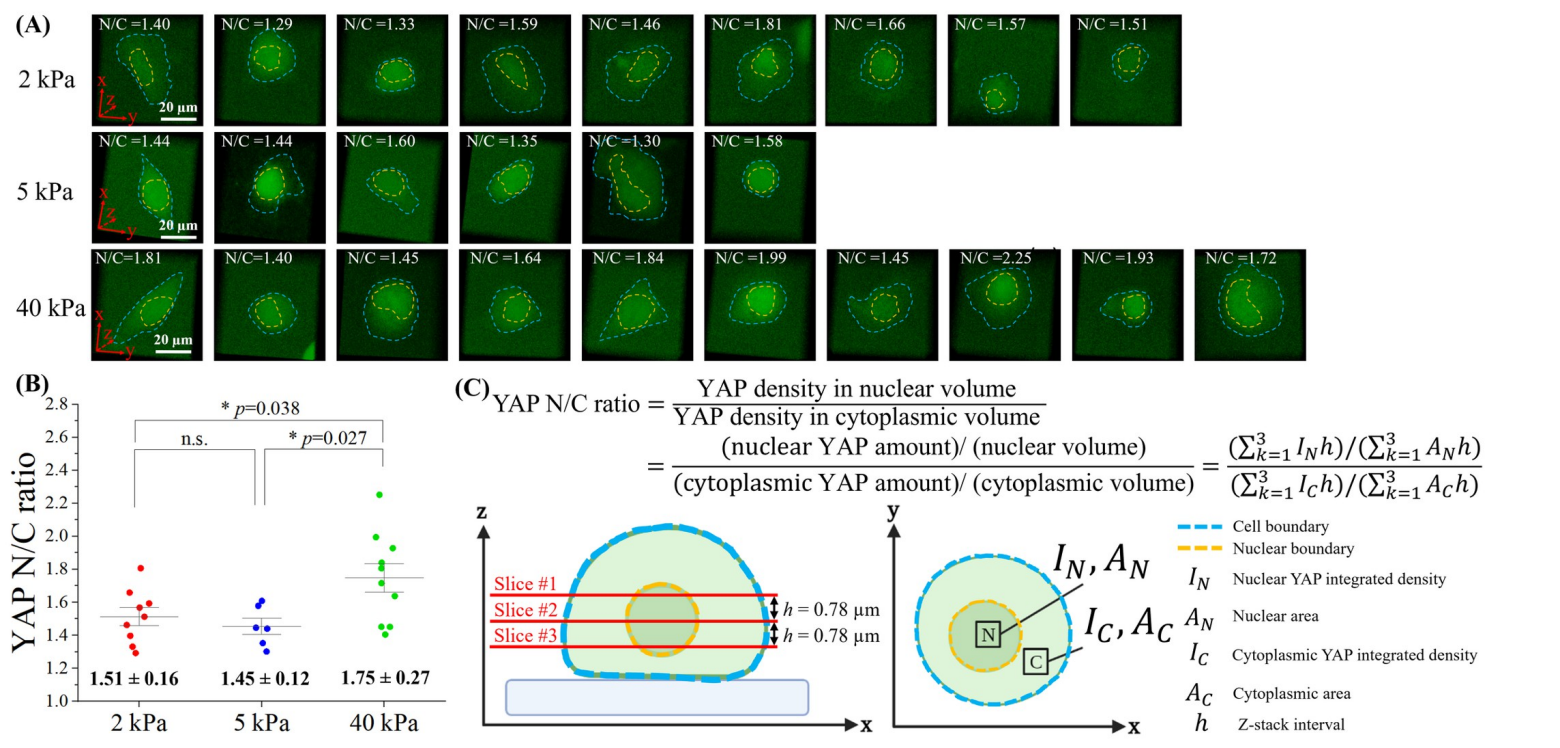
Comparison of the volume-based YAP N/C ratio on hydrogel substrates of different stiffnesses at the 10^th^ hour of the spreading process. **(A)** The 3D reconstruction image of YAP-mNeonGreen2-expressing B2B cells on 2 kPa (n = 9), 5 kPa (n = 6) and 40 kPa (n = 10) at 10^th^ hour. YAP N/C ratio (N/C) for each cell is calculated based on the nuclear volume and cytoplasmic volume. Nuclear boundary and cell boundary are marked by orange and blue dashed line, respectively. **(B)** At 10^th^ hour, volume-based YAP N/C ratio show significant difference between cells on 2 kPa and 40 kPa (* p-value = 0.038). At 10^th^ hour, volume-based YAP N/C ratio show significant difference between cells on 5 kPa and 40 kPa (* p-value = 0.027). **(C)** Volume-based YAP N/C ratio is calculated from 3 slices of the z-stack of YAP images. The interval h between each slice is 0.78 μm. Within each slice, nuclear YAP integrated density (*I_N_*) and cytoplasmic YAP integrated density (*I_C_*) are recorded for an area from nucleus (*A_N_*) and an area from cytoplasm (*A_C_*), respectively.

To verify the capability of AMFIP, we designed and conducted a series of multi-channel and time-lapse image acquisition experiments to elucidate a fundamental cell biology question: can single cells, prior to their full spreading, sense and respond to a soft solid substrate, and if so, how does the interaction depend on the cellular spreading time and the stiffness of the substrate? We employed a CRISPR/Cas9-engineered human epithelial Beas2B (B2B) cell line that expresses mNEonGreen2-tagged Yes-associated protein (YAP) endogenously [21–25]. As a mechano-sensitive protein in cells, YAP, along with transcriptional coactivator with PDZ-binding motif (TAZ), translocates between the nucleus and the cytoplasm depending on the specific mechanical signals received, such as the microenvironment stiffness, shear flow, cell traction, and adhesion [26–29]. The full-spreading cells sense the microenvironment stiffness through their focal adhesion assembly, and subsequently regulate YAP translocation [30–32]. Hence, tracking the spatial-temporal YAP expression in single B2B cells prior to the establishment of full spreading will help answer our question. Specifically, we combined AMFIP and traction force microscopy to co-track the spatial-temporal dynamics of endogenous YAP expression in, cell/nuclear morphology of, and traction force applied by CRISPR/Cas9-engineered B2B cells [33] throughout the process of cell spreading on stiffness-varied hydrogel substrates (Young’s modulus E = 2 kPa, 5 kPa, and 40 kPa). We quantified the ratio of YAP expression in the nucleus/cytoplasm (N/C), spreading areas of nucleus and cell-body, and traction as functions of substrate stiffness (**Figs 3–5**). Interestingly, we found that cells show distinct substrate-dependent distributions of YAP N/C expressions prior to the full spreading of cells (**Figs 6 and 7**).

In summary, our AMFIP program provides a charge-free and hardware-independent solution for multi-functional and time-lapse image acquisition in multidisciplinary biomedical research, including but not limited to optogenetics control, mechanobiology, all-optical electrophysiology imaging, and biomaterials [4, 34–41]. AMFIP coordinates μManager with the Elements platform and other 3^rd^ party software. Its user-friendly GUI allows researchers to flexibly customize and program AMFIP to meet different experimental requirements and discover previously unknown biological phenomena.

## Results

### Graphical User Interface (GUI) of AMFIP

A user-friendly and easily understandable GUI will benefit new users to effectively start their research activities. Based on the Application Program Interface (API) of μManager that prompts users with all inputs of experimental parameters and essential functions available, AMFIP presents such an all-in-one GUI (**Fig 2**) to prescribe and implement multi-functional data acquisition, such as coordination of multiple field-of-views (FOVs), selections of microscope objectives, and modulation of multiple laser channels. Simultaneously, AMFIP preserves full access to other 3^rd^ party software and allows real-time adjustments to achieve customized configurations.

### Design rationale and structure of AMFIP

The workflow of AMFIP follows a model-GUI-controller paradigm that consists of three compartmentalized and interconnected logical components: the model (data), the GUI (**Fig 2**), and the program controller (**Fig 1B**). This paradigm of compartmentalized components enables smooth implementation of new functions by allowing the users to customize any component(s) without interfering with others. For example, users can flexibly change the GUI of AMFIP to a new layout without modifying the model or program controller.

At the beginning of experiments, users input experimental parameters into the GUI (**Fig 2**). Once finished, the controller retrieves the data from the GUI, updates the model, and saves the data to a local hard disk. Alternatively, users can select a previously saved *.JSON configuration file. This selection will restore a previously saved configuration into the input fields of the GUI but will not execute them right away. This feature gives the user the full control to adjust any values of experimental parameters if needed. Alternatively, if a user is entering a new configuration, she/he can choose to save the current configuration for future use. This feature is designed to enable robust repeatability for experiments that take place at different time points and allow other users to replicate and corroborate experiments. Next, the program controller retrieves the inputted data from the model and conducts the data-specified experiments accordingly.

For complex experiments where users want to frequently pause the experimental procedure to adjust for new conditions/samples, such as adding a pharmacological drug into the cells, AMFIP allows users to inform the hardware when to pause and resume the automatic tasks in a user-determined manner. Once manipulation of the conditions/samples is done, users can restore the experiment progress by clicking the “resume” button on the GUI (**Fig 2**). The program will automatically pick up where it left off and continue the pre-defined procedure. AMFIP also has a manual pause function incorporated that researchers can use to pause the tasks whenever they desire. This feature may also serve as an emergency stop.

### Functional synchronization of Nikon NIS-Elements and μManager

Elements is a single and universal software platform that exclusively controls Nikon’s hardware. Currently, Elements is the only commercially available software that controls the Nikon A1R confocal microscope system for 3D image acquisition. However, utilizing its full automatic imaging functionalities requires the purchase of additional Nikon hardware and software. Additionally, the built-in macro inside Elements only achieves automatic image acquisition with limited functionalities [42]. To overcome this restriction, we designed the user-friendly GUI of AMFIP so that users can directly input and compile a sequence of macro commands into a single text field. A macro consists of a sequence of executable commands that utilize a set of pre-defined functions in Elements, e.g., the switch of laser channels, adjustment of laser intensity, or acquisition of 3D z-stack images. This text field generates a *.mac file saved in a specified directory in the local computer. This *.mac file can be loaded into Elements to execute pre-defined functions of the hardware. However, a macro only controls the internal functions of Elements and is incompatible with non-Nikon hardware and software.

To overcome this limitation and to achieve automatic operations, AMFIP enables the functional synchronization of macros and μManager (**Fig 1B**). First, we set a configuration that defines an automatic sequence of motor-stage movements through the GUI. Second, for each FOV to which the motor-stage moves, a script in AMFIP will activate Elements and run a series of commands in the macro editor to execute the experiment-specific functions of the Nikon A1R confocal microscope, such as laser illumination and fluorescent imaging. Upon the completion of image acquisition at each FOV, the macro will generate a blank report and save it into a user-defined file directory. Meanwhile, AMFIP continues checking for this blank report file. Once the program finds this report file, the motor-stage moves to the next FOV and the program deletes the previous file. This process will be automatically repeated in a programmable manner until all pre-selected FOVs are imaged and saved.

### Race-hazard-free coordination between SpinView and μManager

AMFIP can utilize the Blackfly camera that is controlled by SpinView, a GUI provided by FLIR©, to conduct bright-field image acquisition. However, since SpinView cannot directly communicate with other 3^rd^ party software without additional programming, a race hazard between imaging and stage movement may occur during experiment processes. To avoid this potential hazard and achieve safe operations, AMFIP connects SpinView and μManager via Java codes.

Specifically, AMFIP contains a home-built script that controls the keyboard and the cursor of the computer to manipulate SpinView (**Fig 1B**). After μManager executes several automatic operations, such as motor-stage movements and the switch of microscope objectives, the Java code launches SpinView automatically to acquire and save a bright-field image within 1 second. To avoid the condition that the SpinView-regulated image acquisition is perturbed by μManager-regulated stage movement, we pre-allocate a waiting time (usually ~ 2 seconds) during which μManager pauses its operations. This pre-allocated waiting time is sufficient for the operations of SpinView and does not overtly extend the duration of the total experiment. After each image has been captured and saved, μManager resumes its own pre-defined tasks and repeats this cycle till the completion of the experiment. AMFIP is flexible in adding user-compiled scripts either before or after the start of macro in Elements, allowing different experiment requirements to be met in a crosstalk-free manner.

### Connections between MATLAB and μManager for automatic cell detection

AMFIP coordinates with MATLAB to realize the precise detection of cell samples and timely prevention of motor-stage drift during experiments (**Fig 1C**). The first function aims to find all FOVs that contain cells of interest and guide the XY-plane movement of the motor stage to these spots. The mis-match rate (number of detected areas that are not cell divided by number of overall detected areas) is 4.66% ± 0.82%. The second function is to monitor the current coordinates of FOV and to ensure that the cells of interest are captured even if their positions drift during experiments. To achieve frequent and real-time communications between μManager and MATLAB, AMFIP connects the two software via its Java codes.

Similar to the control of SpinView, AMFIP utilizes Java code to control the keyboard and the cursor of the computer to operate on the interface of MATLAB. For precise detection of cell samples, we designed four steps:

1. Use a Blackfly S BFS-U3-70S7M camera to take a bright-field image at 10× magnification under the control of μManager.
2. Launch the MATLAB program by AMFIP to read the image and to distinguish cells from non-cell matters by image processing, such as dilating, smoothing, edge detection, and segmentation.
3. Edit the image, remove incorrect markers on non-cell matters, and add new markers on detection-missed cells on the user-friendly MATLAB GUI.
4. Analyze the edited image by the custom MATLAB programs to generate a text-format list of the coordinates of the selected markers that locate on the cells’ centroid. This text-format list can be read by μManager to guide the movement of the motor-stage on the XY-plane.

For the second function, i.e., monitoring the coordinates in situ, the MATLAB program maintains a specified directory to temporarily save and transfer all captured images one by one. At any given moment, there is only one image file present in this directory. AMFIP constantly monitors this directory, reads this image, and transfers this image to a destination folder. During this process, the MATLAB directory constantly contains only the latest image used to accurately update the current coordinates of FOV. This monitoring function is combined with the AutoFocus functions (Z-axis) provided by the Elements to ensure precise 3D time-lapse imaging for long-term biomedical experiments.

### Differential YAP distribution in B2B cells during the early spreading processes

We applied AMFIP to visualize the spatial-temporal distribution of YAP in single B2B cells during the spreading process. We found that, for B2B cells that have flattened down from the suspension state to the adherent and spreading states on the 2-kPa substrates, YAP expression in the nucleus monotonically decreases in comparison to that in the cytoplasm (n = 10; **Fig 3A, 3B, and 3D**). During the first 10 hours of cell spreading, the average ratio of YAP nucleus/cytoplasm intensity (N/C ratio) changed from 1.97 ± 0.17 to 1.49 ± 0.12 (n = 10; p-value = 0.0022**; photo-bleaching effects not subtracted; **Fig 3D1 and 3D2**; please see **Methods** for analysis algorithms in detail), while the average cell area steadily increased from 708.96 ± 118.71 μm^2^ to 922.30 ± 147.73 μm^2^ (p-value = 0.0920 (ns, i.e., not significant); **Fig 3D1**) and the average nucleus area changed from 222.70 ± 41.35 μm^2^ to 262.37 ± 45.64 μm^2^ (p-value = 0.3220 (ns); **Fig 3D2**). Due to the large heterogeneity of the cell and nucleus spread areas (n = 10), i.e., the initial cell spread areas can vary from 373.01 μm^2^ to 989.35 μm^2^ at 0^th^ hour on 2-kPa substrates, p-values that compare the average cell and nucleus areas at 0^th^ hour (not-full-spreading) vs. 10^th^ hour (full-spreading) are not statistically significant. To validate that the changes in the YAP N/C ratio are independent of the changes in volumes of cytoplasm and nucleus, we calculated the ratio of nucleus-spread-area/cell-spread-area during cell spreading. We found that this ratio approximately changed from 0.27 ± 0.03 to 0.22 ± 0.06 over 10-hr duration (p-value = 0.2422 (ns); **Fig 3D3**), with a linear trend-line slope of -0.0024 that indicates a negligible change in the ratio over time. The statistical and data-fitting results suggest that the sizes of both nucleus and cell body may increase in an approximately proportional manner. Therefore, we reason that the decline of the YAP N/C ratio in single spreading B2B cells on 2-kPa substrates may not be a result of the possible disproportionality between the volume changes of nucleus and cell body.

To estimate the contribution of photobleaching to the decrease of YAP intensity, we examined the single non-spreading B2B cells (n = 5) on 2-kPa substrates in the same experiments (**Fig 3C and 3E**). We found that, during the first 10 hours, the average YAP N/C ratio in non-spreading cells changed from 1.69 ± 0.16 to 1.62 ± 0.09 (p-value = 0.6741 (ns); **Fig 3E1 and 3E2**), while the average cell area changed from 399.49 ± 38.74 μm^2^ to 391.24 ± 21.30 μm^2^ (p-value = 0.8400 (ns); **Fig 3E1**) and the average nucleus area changed from 230.02 ± 43.03 μm^2^ to 225.83 ± 33.60 μm^2^ (p-value = 0.9377 (ns); **Fig 3E2**). These results suggest that (1) photo-bleaching causes only 4% decrease of the YAP fluorescence, which cannot account for the observed 25% decrease of the YAP N/C ratio in spreading cells (**Fig 3D**); and (2) the size of single non-spreading cells nearly do not expand because the ratio of nucleus-spread-area/cell-spread-area remained stable at 0.57 ± 0.07 (p-value = 0.9908 (ns); a linear trend-line slope of -0.0022; **Fig 3E3**). Thus, the non-spreading cells serve as a reasonable control to evaluate the non-photobleaching-induced decline of YAP N/C ratio in spreading cells. Based on the calculated contributions from volume changes and photo-bleaching effects to YAP N/C ratio, we reason that the translocation of YAP from the nucleus to the cytoplasm may cause the decline of YAP N/C ratio in single spreading B2B cells. In single non-spreading B2B cells, fewer YAP, if any, may shuttle from the nucleus to cytoplasm compared to that in single spreading cells. The investigation on mechanisms of why some B2B cells do not spread is ongoing.

We further investigated single B2B cells spreading on stiffer 5-kPa and 40-kPa substrates, respectively (**Figs 4 and 5**). On 5-kPa substrates, during the first 10 hours, the average YAP N/C ratio of single spreading cells changed from 2.39 ± 0.28 to 1.74 ± 0.17 (n = 10; p-value = 0.0057**; photo-bleaching effects not subtracted; **Fig 4E1 and 4E2**), while the average cell spread area increased from 580.96 ± 77.79 μm^2^ to 906.39 ± 245.43 μm^2^ (p-value = 0.0610 (ns); **Fig 4E1**) and the average nucleus spread area rose from 164.85 ± 27.21 μm^2^ to 223.50 ± 43.19 μm^2^ (p-value = 0.0860 (ns); **Fig 4E2**). The average ratio of the nucleus-spread-area/cell-spread-area during the cell spreading process changed from 0.29 ± 0.05 to 0.28 ± 0.05 (p-value = 0.7461 (ns); **Fig 4E3**), with a trend-line slope of 0.0006, indicating that sizes of nucleus and cell body may increase proportionally during cell spreading on 5-kPa substrates. As the control group, we examined the non-spreading cells (n = 5; **Fig 4C and 4F**) from the same experiments. The YAP N/C ratio of non-spreading cells changed from 2.09 ± 0.09 to 2.03 ± 0.12 (p-value = 0.6335 (ns); photo-bleaching effects not subtracted; **Fig 4F1 and 4F2**), while the average cell area increased from 316.12 ± 42.58 μm^2^ to 333.75 ± 40.37 μm^2^ (p-value = 0.7455 (ns); **Fig 4F1**) and the average nucleus area rose from 192.85 ± 15.71 μm^2^ to 218.17 ± 22.10 μm^2^ (p-value = 0.3271 (ns); **Fig 4F2**). The average nucleus-spread-area/cell-spread-area ratio changed from 0.63 ± 0.05 to 0.66 ± 0.03 (p-value = 0.5575 (ns); **Fig 4F3**), with a linear trend-line slope of 0.0051.

On 40-kPa substrates, the average YAP N/C ratio of single spreading B2B cells changed from 2.53 ± 0.32 to 1.93 ± 0.12 in 10 hours (n = 10; p-value = 0.0118 *; photo-bleaching effects not subtracted; **Fig 5A, 5B, 5D1 and 5D2**), while the average cell spread area increased from 988.25 ± 247.34 μm^2^ to 1082.84 ± 207.46 μm^2^ (p-value = 0.6487 (ns); **Fig 5D1**) and the average nucleus spread area increased from 220.70 ± 41.35 μm^2^ to 262.37 ± 45.64 μm^2^ (p-value = 0.3220 (ns); **Fig 5D2**). The average ratio of the nucleus-spread-area/cell-spread-area changed from 0.25 ± 0.05 to 0.26 ± 0.04 (p-value = 0.9317 (ns); **Fig 5D3**), with a trend-line slope of 0.002. As the control, the average YAP N/C ratio of single non-spreading cells from the same experiments changed from 2.16 ± 0.23 to 2.18 ± 0.19 (n = 5; p-value = 0.9440 (ns); photo-bleaching effects not subtracted; **Fig 5C, 5E1 and 5E2**), while the average cell area decreased from 379.36 ± 43.42 μm^2^ to 360.48 ± 37.97 μm^2^ (p-value = 0.7238 (ns); **Fig 5E1**) and the average nucleus area changed from 181.47 ± 39.09 μm^2^ to 234.02 ± 28.37 μm^2^ (p-value = 0.2585 (ns); **Fig 5E2**). The average nucleus-spread-area/cell-spread-area ratio changed from 0.49 ± 0.06 to 0.61 ± 0.04 (p-value = 0.1091 (ns); **Fig 5E3**), with a trend-line slope of 0.0147.

Together, the results suggest that YAP expression in the nucleus of single spreading B2B cells on 2-kPa, 5-kPa, and 40-kPa substrates decreases compared with that in the cytoplasm (**Figs 3D1, 4E1, and 5D1**). Although the YAP N/C ratio in cells decreases on all the three substrates with different stiffness, we identified that the degrees of decrease are different: the YAP N/C ratio decreases more significantly on 2-kPa substrates than that on 5-kPa and 40-kPa substrates. On 2-kPa substrate, the p-value of average YAP N/C ratio in single spreading cells (n = 10) at 0^th^ hour vs. 10^th^ hour is 0.0022 (**; **Fig 3D1 and 3D2**). On 5-kPa substrates, the p-value of average YAP N/C ratio in single spreading cells (n = 10) at 0^th^ hour vs. 10^th^ hour increases to 0.0057 (**; **Fig 4E1 and 4E2**) and is comparatively less statistically significant than that on 2-kPa substrates. On 40-kPa substrate, the p-value of average YAP N/C ratio in single spreading cells (n = 10) at 0^th^ hour vs. 10^th^ hour further increases to 0.0118 (*; **Fig 5D1 and 5D2**) and is even less statistically significant than that on 2-kPa substrates. The results suggest that the changes in the YAP N/C ratio during the first 10 hours of the spreading process are correlated to the substrate stiffness: the change in the YAP N/C ratio declines as the stiffness of the substrates rises. This correlation may indicate that YAP has fewer translocations from the nucleus to the cytoplasm in cells that spread on stiffer substrates during the first 10 hours. The YAP N/C ratios after 10 hours was also measured and persistent mechanotransduction effects on YAP after 10 hours were observed.

To further examine the mechano-sensitivity of these not-full-spreading cells, we compared the p-value of YAP N/C ratio between cells cultured on different substrates (**Fig 6A**; n = 10 cells for each stiffness case). At 10^th^ hour, the p-value of average YAP N/C ratio between 2-kPa and 5-kPa substrates is 0.3103 (ns), and the p-value between 5-kPa and 40-kPa substrates is 0.1637 (ns), while the p-value between 2-kPa and 40-kPa substrates decreases to 0.0262 (*). These statistical results suggest that the difference of YAP N/C distribution at 10^th^ hour becomes greater as the differences of substrate stiffness are larger, i.e., 2 kPa vs. 40 kPa (**Fig 6A**). This trend is found valid at 8 time points of measurements throughout the 10-hour spreading duration, except at 0^th^ and 9^th^ hour (**Fig 6A**). We speculate that these two exceptions are possibly due to the fluctuation of cell dynamics (**Fig 6B and 6C**) because the fluctuation of nucleus size may result in a fluctuating YAP density in the nucleus and influence the trend. Traction applied by cells on substrates seems to not have a role in regulating the fluctuation because we found the magnitude of traction elevated monotonically without fluctuation as cells spread (**Fig 4D**).

We also compared the YAP N/C ratio based on the nuclear volume and cytoplasmic volume of spreading cells cultured on different substrates at 10^th^ hour (**Fig 7A, 7B**; 2-kPa gel (n = 9), 5-kPa gel (n = 6), and 40-kPa gel (n = 10)). The nucleus boundary and the cell boundary are measured on each confocal image slice for the direct calculation of the volume of nucleus and cytoplasm (**Fig 7C**). We found that: At 10^th^ hour, on 2-kPa substrates, the average volume-based YAP N/C ratio is 1.51 ± 0.16. On 5-kPa substrates, the average YAP N/C ratio is 1.45 ± 0.12. On 40-kPa substrates, the average YAP N/C ratio is 1.775 ± 0.27. The p-value of average YAP N/C ratio between 2-kPa and 40-kPa substrates is 0.038 (*) and the p-value between 5-kPa and 40-kPa substrates is 0.027 (*), while the p-value between 2-kPa and 5-kPa substrates is not significant (ns) (**Fig 7B**). These results show that, within 10 hours of culture, single B2B cells develop substrate-stiffness-dependent YAP expression, especially on substrates that have stiffness 2 kPa (or 5 kPa) vs. 40 kPa. Together, we reason that B2B cells, and likely many other types of normal cells, are able to sense, distinguish and respond to different mechanical cues of substrates prior to the establishment of full cell spreading and mature focal adhesion sites − a new phenomenon that has not been reported yet until now.

## Discussion

In this research, we developed and verified an automation program, AMFIP, to overcome the limitations of the latest solutions for automatic image acquisition in biomedical research (**Table 1**). Currently, most manufacturers of optoelectronic hardware provide their own software with limited cost-free opportunities for functional customization and automation [7]_._ These software programs exclusively control manufacturers-specified hardware and require additional financial expenses to coordinate with different microscopy systems [7, 8]_._ In contrast, AMFIP functions as a controlling hub that coordinates multiple software programs to enable modulation of different microscopy systems. Taking our experiments described above as an example, AMFIP can automatically integrate and execute the following functions in a user-defined sequence (**Fig 1B**): (1) AMFIP communicates with μManager to modulate the movement of the XY motor-stage; (2) the Java code of AMFIP activates SpinView to acquire and save bright-field images; (3) the MATLAB program embedded in AMFIP automatically generates a list of coordinates of FOVs based on the acquired bright-field image; and (4) AMFIP reads this list and guides the movement of the XY motor stage based on the coordinates that are constantly updated by the cell detection program.

Moreover, AMFIP provides the long-term utility to the scientific community. Users can customize the GUI and Java code based on their existing hardware environments and experimental requirements. New hardware systems can be easily included into AMFIP either by μManager (μManager-supported-hardware) or the specified Java code (non-μManager-supported-hardware) in the future. The execution of specified Java code embedded in AMFIP functions as a bridge connecting the user’s inputs into AMFIP with the functional and automatic implementation of 3^rd^ party software. Therefore, once users develop the Java-code-enabled coordinations between AMFIP and other 3^rd^ party software, these coordinations activate the existing automatic functions inside these 3^rd^ party software. As a result, users can leverage the existing customizability and automation inside many commercial software programs, such as a macro in Elements, and do not need to compile additional home-built functions starting from scratch, which is potentially time-consuming or technically challenging.

Compared with other software scripting language, such as JOBS, Java is charge-free and has been well developed. Moreover, related controlling software of multiple hardware may support different built-in scripting languages. As a result, it could be difficult to combine different automatic functions from multiple hardware into one experiment. In contrast, through directly sending a series of commands to the computer itself, Java can coordinate various software and thus control different instruments. The source code of AMFIP follows the Java programming rules and the Application Programming Interface of μManager. Any users from experienced programmers to new programmers can easily maintain and modify the code based on the provided instructions of AMFIP on GitHub or available resources from the Internet, i.e., the μManager website and the StackOverflow website.

In addition to our intention to verify the performance of AMFIP, we chose co-tracking the YAP N/C ratio, cell morphology, and traction as an example because mechanistic elucidation of the mechanosensitive YAP/TAZ will deepen our understanding of tissue development, homeostasis, and cancer progression [43, 44]. The nuclear accumulation of YAP/TAZ dominates YAP/TAZ’s active interactions with transcription factors, such as TEADs, RUNXs, and p73, which regulate specific genes expressions that influence cell proliferation, migration, and survival [43, 45, 46]. In particular, recent emerging data suggests that YAP/TAZ show (1) oncogenic effects in most cancer types and (2) tumor-suppressive effects in some cases. In the former case, high protein and mRNA levels of YAP/TAZ in the nucleus are found to be associated with the poor prognosis of cancer patients [32, 47–51]. As the mechanical properties of both tumor cells and tumors microenvironment are dramatically different from those of healthy cells and tissues [52–63], we hypothesize that mechanosensitive YAP/TAZ may distribute aberrantly in the neoplasia niche and affect malignant transformation. Therefore, in this research, we hope to reveal how YAP expression changes as a function of the mechanical environment in a specific context − early cell spreading on 2D planar substrates.

Our results suggest that the YAP N/C ratio in the CRISPR/Cas9-engineered B2B cells may depend on (1) the stiffness of substrates. After 10 hours of culture, the average YAP N/C ratio of spreading single B2B cells is 1.49 on the 2-kPa substrates (n = 10; **Fig 3D1**) and is 1.93 on the 40-kPa substrates (n = 10; p-value = 0.0262*; **Fig 5D1**); and (2) the spreading state of cells, i.e., spreading vs. non-spreading. After 10 hours of culture on 2-kPa substrates, the average YAP N/C ratio changed from 1.97 ± 0.17 to 1.49 ± 0.12 ((n = 10; **Fig 3D1**)) in spreading cells and changed from 1.69 ± 0.16 to 1.62 ± 0.09 in non-spreading cells (n = 5; p-value = 0.3205 (ns); **Fig 3E1**). These results suggest that before the mature establishment of full cell spreading, not-full-spreading B2B cells can sense the different mechanical microenvironments and respond by allowing differential nucleus-to-cytoplasm translocation levels of YAP (**Figs 3–7**). To the best of our knowledge, this phenomenon has not yet been reported by other studies. Research is ongoing in our lab to determine the exact molecular mechanisms that underpin the mechanotransduction processes.

Putting together, AMFIP demonstrates the verified multi-functionality and stability for data acquisition that requires minimum human interferences. AMFIP enables visualization of the real-time interplay (a “dance”) between YAP N/C ratio and substrate mechanics, providing one step towards elucidating YAP/TAZ mechanobiology. These AMFIP-enabled *in vitro* results may inform the future directions of *in vivo* research and ultimately guide the innovation of new cancer therapies.

## Conclusion

In summary, we have developed AMFIP, a hardware-independent and software-based open-source program for automatic data acquisition that is applicable to many microscopy systems and optoelectronic hardware. The intended purposes of AMFIP to study dynamic, multi-faceted, and long-term biological questions are achieved by demonstrating the previously unknown relationship between YAP dynamics and cell mechanics. We think AMFIP is a reliable platform with proven advantages over existing alternatives and validated long-term utility that will benefit the scientific community in multidisciplinary biomedical research [34, 37, 54, 64–71]. The charge-free source code of AMFIP is available to the public on GitHub (link: https://github.com/njheadshotz/AMFIP), and we hope researchers will develop and expand more new functions based on AMFIP to enhance their specific automatic operations in research.

## Materials and methods

### Hardware and software

The protocol described in this peer-reviewed article is published on protocols.io, https://dx.doi.org/dx.doi.org/10.17504/protocols.io.36wgq7ndxvk5/v1 and is included for printing as S1 Protocol with this article. Hardware systems used for experiments include a commercial fluorescent confocal microscope system (Nikon A1R HD25), a monochrome camera (BFS-U3-70S7M-C FLIR©), and a desktop computer that is installed with the 64-bit Microsoft© Windows 10 Pro operating system. The Nikon confocal microscope system consists of multiple components: the Ti2-E inverted microscope, the LU-N4 laser units (405 nm, 488 nm, 561 nm, and 640 nm laser channels), confocal controller, standard fluorescence detector (4 photomultiplier tubes (PMT) and 6 filter cubes), and a scan head (2 galvano scanners and 1 resonant scanner). AMFIP controls the confocal imaging components, such as the laser unit, confocal controller, detectors, and scan head, through activating the Elements. Ti2-E inverted microscope comprises a LED Lamp-house for illumination, motorized XY stage, 6 motorized epi-fluorescence filter turrets, 7 motorized condenser turret, 6 motorized nosepieces, and a Stage joystick. AMFIP controls the Ti2-E inverted microscope by coordinating with μManager.

Confocal 3D image stacks and videos are acquired by the confocal microscope system. The Ti2-E inverted microscope works independently to acquire bright-field images by the monochrome camera. The Dialamp (a white LED equipped on the Ti2-E microscope) serves as a light source for bright-field imaging.

Three software systems are involved to automatically coordinate these devices: (1) SpinView, which controls the BFS-U3-70S7M-C camera; (2) Nikon NIS-Elements, which controls the whole confocal system; and (3) μManager, a 3rd party software, which controls the independent operation of Ti2-E microscope. AMFIP controls all these three software systems through the IntelliJ IDEA platform.

### AMFIP guideline

**Setting up the programming environment.** The following steps show how to set up the software environment to program and implement AMFIP [72].

1. Download and install μManager software from https://micro-manager.org/wiki/Download%20Micro-Manager_Latest%20Release. The latest version, μManager 2.0-gamma, is recommended because of its active development and maintenance.
2. To coordinate μManager with optoelectronic hardware: (1) Connect all needed optoelectronic hardware to a desktop computer and turn on these hardware systems. (2) Add the adaptive drivers called “device adaptor” of the optoelectronic hardware provided by either μManager or the hardware manufacturer into the μManager directory; (3) Go to “Devices->Hardware Configuration Wizard”, check “Create new configuration”, and click “Next”; (4) Find the names of connected hardware in “Available Devices”, click “Add”; (5) A confirmation window pops up. Check their properties and click “OK”; (6) A “Peripheral Devices Setup” window pops up. Select all needed peripheral devices of parent devices (the connected hardware) and click “OK”. These peripheral devices are configured in the list of “Installed Devices”. Click “Next”; (7) Select the default devices and click “Next”; (8) (optional) Set delays for devices without synchronization capabilities and click “Next”; (9) (optional) Define position labels for state devices, such as filters and objectives, and click “Next”; (10) Save the new configuration file, restart μManager, select this configuration file in “Micro-Manager Startup Configuration” and click “OK”.
3. Enable the control of all connected and configured optoelectronic hardware by μManager. For example, we control the Nikon Ti2-E microscope by μManager in our lab. The adaptive driver of the Ti2-E microscope is “Ti2_Mic_Driver.dll ‘‘ located in the “Nikon\Ti2-SDK\bin” of the Ti2 Control software’s directory. This software can be downloaded from https://www.nikon.com/products/microscopesolutions/support/download/software/biological/. Ti2 Control Ver 1.2.0 rather than the latest version is recommended because of its better compatibility with μManager in Microsoft Windows 10 operating system.
4. Download and install IntelliJ IDEA from https://www.jetbrains.com/idea/download/#section=windows for the development of Java-based software.
5. Download and install Java Development Kit (JDK) from https://www.oracle.com/java/technologies/javase-jdk15-downloads.html. JDK 14.0 or higher version is recommended for programming AMFIP.
6. Set up software configuration in IntelliJ to allow developing μManager-based programs. First, open IntelliJ and go to “Settings->Compiler->Annotation Processors”. Check the box of “Enable annotation processing”. Second, go to “Project Structure->Artifacts” and create a JAR(Empty) file. The output directory should be the “mmplugins” folder on the μManager directory. Third, go to “Project Structure->Libraries”, add “mmplugin” and “plugins/Micro Manager” folder from the directory of μManager.
7. Click “add Configuration” and create an application with the following information: “Main class: ij.ImageJ; VM option: -Xmx3000m -Dforce.annotation.index = true; Work directory: μManager directory; Use classpath of module: the name of current project”.
8. Click “Run” in IntelliJ to launch μManager. Click “OK”, and the main interface of μManager appears.

**Use of GUI.** The following steps show how to input pre-defined experimental parameters into the GUI of AMFIP (**Fig 2**) and perform a multi-task experiment.

1. Open μManager, the GUI of AMFIP is under “Plugins->Automation”.
2. Define the number of FOVs to which XY motor-stage moves by clicking “Add Point” or “Remove Point”. Input the coordinates of each FOV into text fields under “Coordinate Panel”. Alternatively, retrieve the saved configurations, i.e., JSON files with a list of previous experimental or pre-defined parameters, including the number/coordinates of FOVs, imaging conditions, and data acquisition parameters.
3. Input a quantitative value into the “Total Experiment Time” text field to define the entire duration of the experiment. Click “Additional Time Configurations”, and input quantitative values into “Start Time”, “Time Interval” and “End Time” for each specified FOV. For each FOV, click “Pause” to program the time when the experiments should automatically stop. The experiments can be resumed by manually clicking “Resume”.
4. Modulate microscope objectives, DiaLamp, and excitation/emission filters for each FOV by inputting pre-defined quantitative values into the three sub-panels below “Coordinate Panel”.
5. Next, click “Save Configuration” to keep a record. Under the submenu of “Devices”, the window of “Device Property Browser” presents a list of quantitative values as a reference, e.g., value “1” for the configuration of microscope objective refers to switching to 10× objective for current FOV.
6. Once all parameters are fed into the GUI, click “Enter” to start a task.
7. In case some unexpected conditions occur during the experimental process, click “Pause” to temporarily stop the experiment. The experiment can be resumed by clicking “Resume”.
8. Once the multi-task experiment is done, to shut down the program, close each window of μManager or directly stop the program from IntelliJ. All the images captured during the experiment are automatically saved into a specified directory.

**Use of home-built Java code to coordinate μManager, Elements, and SpinView.** The following steps show how Java code is applied to automatically coordinate μManager with other software.

1. Open the AMFIP’s Java project in IntelliJ and go to “src”. Scripts are created in “CameraScript” and “ElementsScript”.java files.
2. Go to “Main->Executor”, add two statements: “CameraScript.main()” and “ElementsScript.main()” into “scheduleTaskForAPoint” function. “ElementsScript.main()” activates Elements and runs a pre-defined macro. “CameraScript.main()” activates SpinView to automatically capture and save the bright-field images.
3. To control Elements by Java code, maximize the window of Elements to enclose the window of AMFIP GUI, and enable cursor-based activation of Elements functions. To activate SpinView, place the icon of this software into the taskbar and control the camera by Java code.
4. Initiate step 6 in section 2.2.2. Once XY motor-stage moves, Elements and SpinView are launched and will enable automatic hardware operations following the pre-defined commands in Java code.

### Cell line generation

Generation of endogenously tagged mNeonGreen2_1-10/11_ cell lines was performed in the human bronchial epithelial cell line (Beas2B) as previously described [33]. Briefly, the DNA sequence coding the 11^th^ strand of fluorescence protein mNeonGreen2 is inserted into the gene of interest (i.e., YAP genomic locus) through the CRISPR/Cas9 gene-editing system, and it complements the 1-10^th^ strand of mNeonGreen2 to emit fluorescence and cells with the tagged protein of interest can be collected through fluorescence-activated cell sorting. As a result, mNeonGreen2 is tagged to YAP whenever the cell expresses YAP in the context of its native gene regulatory network. The “knock-in” cell lines are ready to be used without additional exogenous transfections and can be stably maintained for generations. Correct integration of mNeonGreen2_11_ was confirmed by genomic sequencing and by the reduction in fluorescence upon gene knockdown.

### Cell lines maintenance

The Beas2B cell line was maintained in humidified incubators with 5% CO_2_ at 37°C. Beas2B and endogenously tagged derivatives (i.e., the CRISPR/Cas9-engineered cells) were cultured in RPMI-1640 medium supplemented with 10% FBS and penicillin-streptomycin at 100 μg/mL. All cell lines were tested for mycoplasma every 3 months using MycoAlert Mycoplasma Detection Kit (Lonza, Basel, Switzerland). All cells used were <20 passages from thaw.

### Gel preparation

Polyacrylamide (PA or PAA) hydrogel with human fibronectin surface is fabricated before the imaging experiment following the protocol below:

1. Treat the as-received glass coverslip with 3-aminopropyltrymethoxysilane for 7 min at room temperature (24oC).
2. Use deionized (DI) water to rinse the glass coverslip and dry the coverslip for 20 min at 160°C.
3. Treat the glass coverslip with 0.5% glutaraldehyde for 30 min and rinse by DI water.
4. Mix acrylamide solution, N,N′-methylenebisacrylamide (bis) solution, and fluorescent beads solution in 10 mM HEPES-buffered saline. Use 10% (w/v) ammonium persulfate solution and N,N,N′,N′-tetramethylethylenediamine (TEMED) as initiator of polymerization. Change the percentage of each component to achieve desired mechanical stiffness of PA hydrogels following the established protocols [39, 59].
5. After 35 min, peel the glass coverslip from the PA hydrogel and wash the hydrogel with 50 mM HEPES-buffered saline twice (5 min each time).
6. Treat the PA hydrogel surface with hydrazine-hydrate solution for 6 hours.
7. Rinse the PA hydrogel by acetic acid for 30 min. Remove acetic acid and rinse by phosphate buffered saline (PBS) for 30 min.
8. Oxidize as-received fibronectin solution (50 μg/mL in PBS) by sodium periodate for 30 min.
9. Coat the oxidized fibronectin solution on the surface of PA hydrogel and wait for 35 min.
10. Add PBS to immerse the PA hydrogel and store at 4°C. Cover all the petri dishes that contain the hydrogels with Aluminum foils to avoid any light exposure to the hydrogels.
11. Apply the atomic force microscopy (AFM) and silicon-nitride cantilevers that have a spring constant *k* = 148.14 pN×nm^-1^ (Veeco) to perform indentation on the PA hydrogels in aqueous conditions. Characterize the mechanical properties, e.g., the Young’s modulus, of the PA hydrogel based on the obtained force vs. indentation curves using Hertz theory. The detailed protocol is published [59].

### Cell imaging

The following steps show how to achieve a multi-functional and time-lapse image acquisition using AMFIP to observe traction dynamics and YAP dynamics of the YAP-B2B cell line.

1. Turn on the Nikon A1R confocal microscope system following a specific sequence: LU-N4 laser units, the confocal controller, the Ti2-E microscope controller, and the Ti2-E inverted microscope.
2. In the Ti2-E inverted microscope, switch to 10× objective and the light-path on the right side for BF imaging to identify the cells of interest. Using the 10× magnification, open μManager and move XY motor-stage by joystick to find appropriate FOVs containing both single and multiple adjacent cells that grow well on the substrate. For each 10× FOV, switch to 40× objective, adjust XY motor-stage again to have the specified FOVs in the center, and record coordinates of selected FOVs.
3. Input these coordinates and pre-defined experimental parameters into the GUI of AMFIP. For the experiment described in Results F, 40× objective and 5% of DiaLamp intensity are applied.
4. Launch Elements, open the FITC channel and switch to the resonant scanner for fast-speed imaging. In the experiment described in Results. F, fluorescent images captured in the FITC channel display the YAP dynamics of B2B cells that express YAP: mNeonGreen2_1-10/11_. Slowly adjust the knob of the Z-plane and record the highest and the lowest Z position to form a z-stack that covers the overall z-height of cells that start adhering to the substrate.
5. Open the DAPI channel and close the FITC channel. In the experiment described in Results F, fluorescent images captured in the DAPI channel present displacement of beads that can be used to calculate traction dynamics. Next, slowly adjust the knob to change the Z-plane and record the highest and the lowest Z position to generate a z-stack covering the interface between the top surface of the substrate and cell bottom.
6. In the macro editor, to generate z-stack images for both laser channels, write specified commands and input (a) 4 quantitative values collected from previous steps and (b) an appropriate step size of z-plane to generate sufficient numbers of frames for a 3D z-stack image. Next, switch back to galvano scanner for high-resolution imaging. To avoid photobleaching of fluorophore and capture images with low noise, we set the exposure time to 4 seconds for the above experiments.
7. Complete the rest of the commands in a macro to achieve the following functions in sequence:
  - Close DiaLamp and switch to the light-path on the left side for fluorescent imaging.
  - Switch to the FITC laser channel and start z-stack image acquisition.
  - Switch to the DAPI laser channel and start a z-stack image acquisition of beads.
  - Save the two z-stack images to a specified directory for data analysis.
  - Switch back to the light-path on the right side and turn on the DiaLamp that allows μManager to take a bright-field image.
8. Back to the GUI of AMFIP. To avoid photobleaching, set the time interval for image acquisition of each FOV to 30 minutes. Next, set the total duration of the experiment to 12 hours or above. Next, click “Enter” to start the imaging process.
9. For each time interval (i.e., 30 minutes for the experiment described here), AMFIP automatically executes the following operations in sequence:
  - Move XY motor-stage to each pre-selected FOV.
  - Take and save separate z-stack images for FITC and DAPI channels through Elements.
  - Capture and save a bright-field image through μManager.
  - After all imaging processes are completed in one FOV, AMFIP automatically instructs XY motor-stage to move to the next FOV and repeat the previous operations.
10. Once the experiment is finished, shut down the Nikon A1R confocal microscope system following a specific sequence: the Ti2-E inverted microscope, the Ti2-E microscope controller, the confocal controller, and the LU-N4 laser units.

Each imaging condition of multi-channel images is listed below:

- Bright-field image: magnification: 40×; DiaLamp intensity: 10%; exposure time: 14ms.
- Z-stack image of DAPI channel: magnification: 40×; laser intensity: 30%; gain of photomultiplier tube: 125; exposure time: 4s; step size: 5 μm; the range of Z-plane: 10 μm.
- Z-stack image of FITC channel: magnification: 40×; laser intensity: 30%; gain of photomultiplier tube: 70; exposure time: 4s; step size: 2 μm; the range of Z-plane: 30~40 μm.
- Z-stack image of FITC channel for 3D imaging: magnification: 40×; laser intensity: 30%; gain of photomultiplier tube: 70; exposure time: 4s; step size: 0.78 μm; the range of Z-plane: 30~40 μm.

Bright-field image and fluorescence image can be acquired separately or together. If both images are captured in turn, the maximum frame rate of AMFIP is 20 sec/frame considering the time for switching light path. If single type of image is captured, the maximum frame rate could reach to 0.5 sec/frame. This value also depends on the exposure time in specific experiment settings.

### Images processing and analysis

The ratio of fluorescence intensity between nucleus and cytoplasm (N/C ratio) is widely used in live-cell experiments for the analysis of protein dynamics and cell functions. To examine the relationship between YAP N/C ratio and cell-area/nucleus-area ratio, we analyzed time-lapse images of single spreading cells and non-spreading cells on different substrates (**Figs 3–6**) by using Fiji ImageJ to process the acquired confocal and bright-field images (**S1 Protocol**) [72, 73].

### Statistics analysis

We applied a student’s t-test to evaluate the statistical significance of the data (shown in **Figs 3D, 3E, 4E, 4F, 5D, 5E, 6A & 7B**). To execute this t-test, we input two different data groups for comparison. The calculation generates a p-value that indicates whether there is a significant difference between the two data groups (i.e., p ≤ 0.001: [***]; 0.001 < p ≤ 0.01: [**]; 0.01 < p ≤ 0.05: [*]; 0.05 < p: ns). For both spreading and non-spreading cells, we formed four data groups: YAP N/C ratio, cell area, nucleus area, and nucleus-area/cell-area ratio. We compared the data of each group at 10^th^ hour with the data of the same group at 0^th^ hour. The calculated p-value indicates whether there is a significant statistical difference during the first 10 hours of cell spreading. Next, we applied the t-test to compare the average YAP N/C ratio of single spreading cells on different substrates over time.

### Traction force microscopy

We conducted traction force microscopy experiments following the established protocol [39, 74–77]. Briefly, first, we combined two images containing the view of fluorescent beads on substrates’ surfaces into an image stack. The first image is the reference, i.e., cells after dissolution. The second image is the deformed view, i.e., cells before dissolution. Second, we applied Particle Image Velocimetry (PIV; ImageJ plugin) to measure the displacement field. Third, based on the displacement field, we used the FTTC ImageJ plugin to operate the Fourier Transform Traction Cytometry method and calculated the traction force field [78].

## Supporting information

S1 Protocol (PDF)
