# Supplementary material for: Automatic Multi-functional Integration Program (AMFIP) towards all-optical mechano-electrophysiology interrogation
Source: PLoS One. 2022 Jul 28;17(7):e0266098. doi: 10.1371/journal.pone.0266098 (PMC9333221; doi:10.1371/journal.pone.0266098)
Supplement: S1 Protocol — (PDF) [file pone.0266098.s001.pdf]

# S1 Protocol

## AMFIP setup

### Setting up the programming environment

The following steps show how to set up the software environment to program and implement AMFIP:

1. Download and install µManager software from https://micro-manager.org/wiki/Download%20Micro-Manager_Latest%20Release. The latest version, µManager 2.0-gamma, is recommended because of its active development and maintenance.
2. To coordinate µManager with optoelectronic hardware: (1) Connect all needed optoelectronic hardware to a desktop computer and turn on these hardware systems. (2) Add the adaptive drivers called "device adaptor" of the optoelectronic hardware provided by either µManager or the hardware manufacturer into the µManager directory; (3) Go to "Devices->Hardware Configuration Wizard", check "Create new configuration", and click "Next"; (4) Find the names of connected hardware in "Available Devices", click "Add"; (5) A confirmation window pops up. Check their properties and click "OK"; (6) A "Peripheral Devices Setup" window pops up. Select all needed peripheral devices of parent devices(the connected hardware) and click "OK". These peripheral devices are configured in the list of "Installed Devices". Click "Next"; (7) Select the default devices and click "Next"; (8) (optional) Set delays for devices without synchronization capabilities and click "Next"; (9) (optional) Define position labels for state devices, such as filters and objectives, and click "Next"; (10) Save the new configuration file, restart

µManager, select this configuration file in “Micro-Manager Startup Configuration” and click “OK”.

3. Enable the control of all connected and configured optoelectronic hardware by µManager. For example, we control the Nikon Ti2-E microscope by µManager in our lab. The adaptive driver of the Ti2-E microscope is “Ti2_Mic_Driver.dll ‘’ located in the “Nikon\Ti2-SDK\bin” of the Ti2 Control software’s directory. This software can be downloaded from https://www.nikon.com/products/microscopesolutions/support/download/software/biological/. Ti2 Control Ver 1.2.0 rather than the latest version is recommended because of its better compatibility with µManager in Microsoft Windows 10 operating system.
4. Download and install IntelliJ IDEA from https://www.jetbrains.com/idea/download/#section=windows for the development of Java-based software.
5. Download and install Java Development Kit (JDK) from https://www.oracle.com/java/technologies/javase-jdk15-downloads.html. JDK 14.0 or higher version is recommended for programming AMFIP.
6. Set up software configuration in IntelliJ to allow developing µManager-based programs. First, open IntelliJ and go to “Settings->Compiler->Annotation Processors”. Check the box of “Enable annotation processing”. Second, go to “Project Structure->Artifacts” and create a JAR(Empty) file. The output directory should be the “mmplugins” folder on the µManager directory. Third, go to “Project Structure->Libraries”, add “mmplugin” and “plugins/Micro Manager” folder from

the directory of μManager.

7. Click "add Configuration" and create an application with the following information: "Main class: ij.ImageJ; VM option: -Xmx3000m -Dforce.annotation.index=true; Work directory: μManager directory; Use classpath of module: the name of current project".
8. Click "Run" in IntelliJ to launch μManager. Click "OK", and the main interface of μManager appears.

## Use of GUI

The following steps show how to input pre-defined experimental parameters into the GUI of AMFIP and start a multi-task experiment.

1. Open μManager, the GUI of AMFIP is under "Plugins->Automation".
2. Define the number of FOVs to which XY motor-stage moves by clicking "Add Point" or "Remove Point". Input the coordinates of each FOV into text fields under "Coordinate Panel". Alternatively, retrieve the saved configurations, i.e., JSON files with a list of previous experimental or pre-defined parameters, including the number/coordinates of FOVs, imaging conditions, and data acquisition parameters.
3. Input a quantitative value into the "Total Experiment Time" text field to define the entire duration of the experiment. Click "Additional Time Configurations", and input quantitative values into "Start Time", "Time Interval" and "End Time" for each specified FOV. For each FOV, click "Pause" to program the time when the experiments should automatically stop. The experiments can be resumed by manually clicking "Resume".
4. Modulate microscope objectives, DiaLamp, and excitation/emission filters for each

FOV by inputting pre-defined quantitative values into the three sub-panels below "Coordinate Panel".

5. Next, click "Save Configuration" to keep a record. Under the submenu of "Devices", the window of "Device Property Browser" presents a list of quantitative values as a reference, e.g., value "1" for the configuration of microscope objective refers to switching to 10× objective for current FOV.
6. Once all parameters are fed into the GUI, click "Enter" to start a task.
7. In case some unexpected conditions occur during the experimental process, click "Pause" to temporarily stop the experiment. The experiment can be resumed by clicking "Resume".

## Use of home-built Java code to coordinate μManager, Elements, and SpinView

The following steps show how Java code is applied to automatically coordinate μManager with other software.

1. Open the AMFIP's Java project in IntelliJ and go to "src". Scripts are created in "CameraScript" and "ElementsScript" .java files.
2. Go to "Main->Executor", add two statements: "CameraScript.main()" and "ElementsScript.main()" into "scheduleTaskForAPoint" function. "ElementsScript.main()" activates Elements and runs a pre-defined macro. "CameraScript.main()" activates SpinView to automatically capture and save the bright-field images.
3. To control Elements by Java code, maximize the window of Elements to enclose the window of AMFIP GUI, and enable cursor-based activation of Elements

functions. To activate SpinView, place the icon of this software into the taskbar and control the camera by Java code.

4. Initiate step 6 in section 2.2.2. Once XY motor-stage moves, Elements and SpinView are launched and will enable automatic hardware operations following the pre-defined commands in Java code.

# Cell imaging

The following steps show how to achieve a multi-functional and time-lapse image acquisition using AMFIP to observe traction dynamics and YAP dynamics of the YAP-B2B cell line.

1. Turn on the Nikon A1R confocal microscope system following a specific sequence: LU-N4 laser units, the confocal controller, the Ti2-E microscope controller, and the Ti2-E inverted microscope.
2. In the Ti2-E inverted microscope, switch to 10× objective and the light-path on the right side for BF imaging to identify the cells of interest. Using the 10× magnification, open μManager and move XY motor-stage by joystick to find appropriate FOVs containing both single and multiple adjacent cells that grow well on the substrate. For each 10× FOV, switch to 40× objective, adjust XY motor-stage again to have the specified FOVs in the center, and record coordinates of selected FOVs.
3. Input these coordinates and pre-defined experimental parameters into the GUI of AMFIP. For the experiment described in Results F, 40× objective and 5% of DiaLamp intensity are applied.
4. Launch Elements, open the FITC channel and switch to the resonant scanner for

fast-speed imaging. In the experiment described in Results. F, fluorescent images captured in the FITC channel display the YAP dynamics of B2B cells that express YAP: mNeonGreen2$_{1-10/11}$. Slowly adjust the knob of the Z-plane and record the highest and the lowest Z position to form a z-stack that covers the overall z-height of cells that start adhering to the substrate.

5. Open the DAPI channel and close the FITC channel. In the experiment described in Results F, fluorescent images captured in the DAPI channel present displacement of beads that can be used to calculate traction dynamics. Next, slowly adjust the knob to change the Z-plane and record the highest and the lowest Z position to generate a z-stack covering the interface between the top surface of the substrate and cell bottom.
6. In the macro editor, to generate z-stack images for both laser channels, write specified commands and input (a) 4 quantitative values collected from previous steps and (b) an appropriate step size of z-plane to generate sufficient numbers of frames for a 3D z-stack image. Next, switch back to galvano scanner for high-resolution imaging. To avoid photobleaching of fluorophore and capture images with low noise, we set the exposure time to 4 seconds for the above experiments.
7. Complete the rest of the commands in a macro to achieve the following functions in sequence:
   a. Close DiaLamp and switch to the light-path on the left side for fluorescent imaging.
   b. Switch to the FITC laser channel and start z-stack image acquisition.
   c. Switch to the DAPI laser channel and start a z-stack image acquisition of

beads.

d. Save the two z-stack images to a specified directory for data analysis.

e. Switch back to the light-path on the right side and turn on the DiaLamp that allows μManager to take a bright-field image.

8. Back to the GUI of AMFIP. To avoid photobleaching, set the time interval for image acquisition of each FOV to 30 minutes. Next, set the total duration of the experiment to 12 hours or above. Next, click "Enter" to start the imaging process.
9. For each time interval (i.e., 30 minutes for the experiment described here), AMFIP automatically executes the following operations in sequence:

   a. Move XY motor-stage to each pre-selected FOV.

   b. Take and save separate z-stack images for FITC and DAPI channels through Elements.

   c. Capture and save a bright-field image through μManager.

   d. After all imaging processes are completed in one FOV, AMFIP automatically instructs XY motor-stage to move to the next FOV and repeat the previous operations.

Each imaging condition of multi-channel images is listed below:

a. Bright-field image: magnification: 40×; DiaLamp intensity: 10%; exposure time: 14ms.

b. Z-stack image of DAPI channel: magnification: 40×; laser intensity: 30%; gain of photomultiplier tube: 125; exposure time: 4s; step size: 5 μm; the

range of Z-plane: 10 μm.

c. Z-stack image of FITC channel: magnification: 40×; laser intensity: 30%; gain of photomultiplier tube: 70; exposure time: 4s; step size: 2 μm; the range of Z-plane: 30~40 μm.

d. Z-stack image of FITC channel for 3D imaging: magnification: 40×; laser intensity: 30%; gain of photomultiplier tube: 70; exposure time: 4s; step size: 0.78 μm; the range of Z-plane: 30~40 μm.

## Images processing and analysis

The following steps show how to use Fiji ImageJ to process and analyze the acquired confocal and bright-field images:

1. Launch Fiji ImageJ, open all bright-field images from one FOV and concatenate them into a stack. Next, open the confocal z-stack image of YAP from the same FOV.
2. Scale down and fit the size of the bright-field image stack to the size of the confocal image. Go to "Image->Overlay->Add Image", select the bright-field image as "image to add". Next, Set the opacity value to 50 and click "OK". This process allows us to overlap the bright-field image on the confocal image.
3. Locate the cell being examined and align the cell boundary in the bright-field image with similar shaped YAP fluorescence in the confocal image.
4. Go to "Analyze->Set Measurement", and check functions: "Area", "Mean grey value", and "Integrated density". For the experiment described in Results F, "Area" measures the area of the selected region of interest (ROI)

from the image being processed. “Mean grey value” presents the relative YAP density of the ROI in this experiment. “Integrated density” displays the relative YAP intensity of the ROI, which is the product of the data from “Area” and “Mean grey value”.

5. Choose “Freehand selection” on the main interface of ImageJ, first carefully select the ROI of the nucleus of the examined cell and click “Analyze->Measure”. A “Results” window pops up. Second, select a new ROI of the cell boundary of the same cell and redo “Analyze->Measure”.
6. Repeat step 5 for every frame of the confocal image stack. Next, copy the data from the “Results” window and paste it into an excel file for data analysis.
7. To determine the YAP density in the cytoplasm, first calculate the difference between the nucleus area (AN) and the cell-body area (CN) representing the cytoplasm area. Second, calculate the difference between the integrated density of the cell-body (IC)  and the nucleus (IN). This value represents the relative intensity of YAP in the cytoplasm. Third, calculate the YAP density by dividing the YAP intensity in the cytoplasm by the cytoplasm area.
8. To calculate the YAP N/C ratio, divide the YAP density of the nucleus by the YAP density of the cytoplasm, i.e., N/C ratio = (IN /AN)/(( IC-IN)/( AC-AN)).
9. Repeat step 1 to step 8 for all cells being studied. Next, form and analyze multiple line charts based on the processed data.
